# Attention Deficit Hyperactivity Disorder in the Light of the Epigenetic Paradigm

**DOI:** 10.3389/fpsyt.2015.00126

**Published:** 2015-09-17

**Authors:** Viviane Schuch, Daniel Augusto Utsumi, Thaís Virgínia Moura Machado Costa, Leslie Domenici Kulikowski, Mauro Muszkat

**Affiliations:** ^1^Núcleo de Atendimento Neuropsicológico Infantil Interdisciplinar (NANI), Centro Paulista de Neuropsicologia, Departamento de Psicobiologia da Universidade Federal de São Paulo – UNIFESP, São Paulo, Brazil; ^2^Laboratório de Citogenômica, LIM 03, Departamento de Patologia, Faculdade de Medicina da Universidade de São Paulo, São Paulo, Brazil

**Keywords:** ADHD, executive functions, emotional self-regulation, DESR, genetic factors, environmental factors, epigenetics

## Abstract

Attention deficit hyperactivity disorder (ADHD) is a highly prevalent neurodevelopmental disorder characterized by a definite behavioral pattern that might lead to performance problems in the social, educational, or work environments. In the Diagnostic and Statistical Manual of Mental Disorders, Fifth Edition, the symptoms of ADHD were restricted to those associated with cognitive (attention deficit) and behavioral (hyperactivity/impulsivity) deficits, while deficient emotional self-regulation, a relevant source of morbidity, was left out. The etiology of it is complex, as its exact causes have not yet been fully elucidated. ADHD seems to arise from a combination of various genetic and environmental factors that alter the developing brain, resulting in structural and functional abnormalities. The aim of this paper was to review epigenetics and ADHD focused on how multidimensional mechanisms influence the behavioral phenotype.

## Attention Deficit Hyperactivity Disorder

Attention deficit hyperactivity disorder (ADHD) is a neurodevelopmental disorder characterized by a definite behavioral pattern that might impair the affected individuals’ performance in the social, educational, or work environments. According to epidemiological studies, ADHD affects approximately 3–6% of schoolchildren worldwide and continues into adolescence and adulthood in most cases (50–80%) (1–4). This high prevalence is a cause of concern because ADHD has negative impacts on all neurodevelopmental areas and on the psychosocial interactions of the affected individuals. The risk might be increased when symptoms, such as aggressiveness and irritability, or comorbidities, such as conduct disorder or oppositional defiant disorder, are present (5, 6).

According to Diagnostic and Statistical Manual of Mental Disorders, Fifth Edition (DSM-V), the symptoms of ADHD manifest as inappropriate levels of inattention, hyperactivity, and impulsivity, including behaviors, such as inability to pay attention to details, difficulty organizing tasks, and activities, and restlessness or inability to remain sitting in appropriate situations (7). There is no biological marker for ADHD. Clinical diagnosis is established according to the criteria listed in DSM-V, based on the child’s clinical history and the repercussion of the behavioral symptoms on their network of relationships (8). Clinical diagnosis is complemented by neuropsychological assessment, which allows establishing the magnitude of the deficits and detecting impairments in other functional areas.

Neuroimaging studies indicate that ADHD is a result of abnormal anatomical functioning and connectivity throughout fronto-striatal, fronto-temporal, fronto-parietal, and/or fronto-striato-parieto-cerebellar circuitry. In addition to circuitry, specific structures and areas of the brain have also received attention and include, among others, the prefrontal cortex, anterior cingulate cortex, caudate, globus pallidus, parietal regions, temporal regions, corpus callosum, splenium, cerebellar vermis, and cerebellum (9). Neuropsychological studies combined with brain imaging methods have detected functional alterations in neural networks in specific brain areas related with executive functions (EFs) (10, 11).

### Executive functions and emotional self-regulation

In recent years, studies suggested that executive dysfunction is the main deficit in ADHD (10). The EFs are a set of cognitive skills, associated with prefrontal cortex functions, that allow individuals to orient and shape their behavior to attain goals (12–15). Currently, the EFs are divided into cold and hot. The cold EFs are considered to be purely cognitive and used in tasks that require handling abstract concepts, such as attention, working memory, planning, cognitive flexibility, alternation, and inhibition. These EFs are mainly related with dorsolateral prefrontal cortex function (11, 16). In turn, the hot EFs are used in tasks that demand motivation and affect and are associated with the orbitofrontal and ventromedial cortices (11, 16–18). Although neuropsychological assessment of EFs provides a significant contribution to the understanding of ADHD, most tests are performed in artificial settings, and the results often do not reflect the deficits exhibited by the affected children in their everyday lives (19). Scales, such as the child behavior checklist (CBCL), are currently being used to detect behavioral deviations in the different settings of the life of a child, such as school and home (20). In addition to provide quantitative measures, CBCL allows for the assessment of some features of the hot EFs, including emotional self-regulation (21).

Self-regulation might be described as an ability that allows individuals to plan, choose between options, control impulses, inhibit undesired thoughts, and regulate their social behavior. These characteristics are quite close to the definition of the hot EFs themselves. Therefore, not by chance, Barkley defined the EFs as self-regulation. Several studies suggest that ADHD might result from deficient emotional self-regulation (DESR) (22). DESR involves deficient self-regulation of the physiological excitation caused by strong emotions, difficulties inhibiting inadequate behaviors in response to positive or negative emotions, problems reorienting attention after a strong emotion, and disorganization of coordinated behavior in response to emotional activation. DESR traits include low tolerance to frustration, impatience, irritability, and easy excitation of emotional reactions (21, 23, 24). A longitudinal study conducted with 177 children with ADHD and 204 children without it detected strong correlations between ADHD and DESR, which is defined as a *t*-score >180 but <210 on the attention, aggression, and anxiety/depression subscales of the CBCL. Approximately 44% of the children with ADHD exhibited a positive CBCL-DESR profile versus 2% of the children without ADHD (21). Presence of a positive CBCL-DESR profile was associated with higher rates of anxiety and disruptive behavior disorders and with greater impairments of the emotional, interpersonal, and psychosocial functions, even after adjustment for comorbidities (24). Those studies show that the presence of a positive CBCL–DESR profile might help to identify the subgroup of children with ADHD at higher risk of individual and social impairment. As a result of deficient self-regulation, some psychological processes and functions, including working memory, internalization of expression, the sense of time, and goal-directed behavior, do not develop in a satisfactory manner (5). From that perspective, and thus different from the definition in the DSM-V, attention deficit might be a secondary aspect in ADHD.

## Etiology of ADHD

The etiology of ADHD has been the subject of several studies. Although the number of such studies is large, the exact causes of ADHD have not yet been fully elucidated. ADHD seems to result from a combination of genetic and environmental factors that alter the developing brain, resulting in structural and functional abnormalities (25).

### Genetic factors

Several genetic linkage and association studies have been conducted in recent years to identify candidate genes related to ADHD (26). A recent meta-analysis that included seven linkage studies (27–35) confirmed the importance of a region on chromosome 16 (16q21–16q24). In addition, 10 genomic regions on 8 chromosomes (5, 6, 7, 8, 9, 15, 16, and 17) were associated with ADHD (36). However, none of the studies led to the identification of genes significantly related to ADHD but rather explained small parts of their genetic contribution to the disease. Even association studies of the entire genome (GWAS – genome-wide association study) were unable to identify any significant associations and described a rather limited superposition of genes (26).

Despite the small number of truly significant findings involving genes, it is known that genetic factors strongly influence ADHD (4). This phenomenon is mainly evidenced by the high heritability, which has been estimated at 76% based on studies with families and adopted children (26, 36–38). Those studies confirmed that ADHD has a genetic predisposition because the risk of disease was higher among the biological siblings of children with ADHD compared to the adopted ones. In addition, those studies showed that the risk of adopted relatives of children with ADHD was similar to that of the relatives of the children in the control group (4, 37, 39). However, although the literature indicates that ADHD is a highly heritable condition, no gene could yet be named as necessary or sufficient for the disease to occur. Thus, the investigation of susceptibility genes is the subject of many studies.

The main targets of such research are the genes that encode components of the dopaminergic, noradrenergic, and serotonergic systems (40–42). Dopamine is a catecholamine neurotransmitter involved in the control of motion, learning, mood, emotions, cognition, sleep, and memory. It is a natural precursor of epinephrine and norepinephrine, other catecholamines with stimulant actions on the central nervous system. Dopamine dysregulation is associated with several neuropsychiatric disorders, for which reason it was the target of many molecular studies on ADHD (41–43). The dopamine active transporter 1 (DAT1, also known as SLC6A3) gene was the first to be investigated, as this transport protein is involved in the modulation of the effects of the stimulating drugs commonly used for ADHD treatment (44, 45). Dopamine receptors, especially DRD4 and DRD5, are also strongly associated with ADHD development (46–48). Few molecular studies with genes related to the noradrenergic system have been performed. Such studies mainly focused on the gene that encodes dopamine-beta-hydroxylase (DβH), which catalyzes the degradation of dopamine into norepinephrine, with direct effects on the total level of dopamine in the brain (49). The serotonergic system might also participate in ADHD etiology, in particular the serotonin receptor (HTR1B) and transporter (SCL6A4) genes (50).

There is emerging evidence that GABAergic and glutamatergic system play important roles in the pathophysiology of ADHD. Children with ADHD appear to have more than twice the level of glutamate, which is an excitatory neurotransmitter for neuronal cells, and low level of gamma-aminobutyric acid (GABA), which acts as an inhibitory molecule to inhibit stimulated neuronal cells (51). Genetic studies have reported an association between ADHD and a number of glutamate receptor gene variants (GRM1, GRM5, GRM7, and GRM8) (52). Furthermore, some animal models suggest that altered glutamate regulation of the dopamine system underpins the functional deficits associated with ADHD (53).

Some evidence points to an association between the synaptosomal-associated protein 25 (SNAP-25) gene and ADHD. SNAP-25 is one the proteins of the presynaptic plasma membrane and is highly and specifically expressed in neurons, being crucial for the fusion of synaptic vesicles and neurotransmitter release. A study showed that rats with mutations in that gene exhibited marked hyperactivity and other behaviors consistent with ADHD phenotype (54). Subsequent studies have suggested that mutations in the SNAP-25 gene might predispose individuals to ADHD (55, 56). Monoamine oxidase A (MAO-A) participates in the degradation of components of the dopaminergic, adrenergic, and serotonergic systems and thus has been suggested as a strong candidate to account for ADHD susceptibility (57). One additional candidate is brain-derived neurotrophic factor (BDNF), a member of the neurotrophins family, which is involved in some neurodevelopmental processes, including the survival and differentiation of dopaminergic neurons in the developing brain and glutamate-dependent neuronal plasticity in adults. Evidence gathered in animal studies shows that the expression of BDNF increases in the amygdala, piriform cortex, and hypothalamus of rats following repeated administration of amphetamine, which suggests that BDNF participates in the psychostimulant response. In addition, reduction of the central serotonergic activity has been associated with poor impulse regulation (a key-feature of ADHD) in youths, adults, and animals (58).

### Environmental factors

The environmental factors that are epidemiologically associated with ADHD include psychosocial adversities, maternal mental disorders, violence, stress, smoking, and drinking alcohol in the prenatal period and childhood. A longitudinal study conducted in Brazil by Pires and colleagues sought to correlate the family environment and pregnancy with ADHD diagnosis in children and the symptoms described by various informants (mothers and teachers). These authors found that family dysfunction, lack of social support for mothers, adverse life events, and disagreements in the course of pregnancy were associated with mother-reported ADHD (59). ADHD is frequently associated with exposure to violence in intrauterine life and childhood, including domestic violence (60). Class and colleagues conducted a large population-based study in Switzerland and found that exposure of pregnant women to stress in the third trimester of pregnancy increased the risk of ADHD (61). Premature birth is also associated with increased risk of ADHD. Not only extremely (23–28 weeks) but also moderately (33–36 weeks) premature birth increases the risk of ADHD, most likely due to the degree of immaturity at birth. Social adversities, expressed as a low maternal educational level, also influence the risk of ADHD among children who were moderately premature (62). The risk of ADHD is also higher among children who were too small or too large for their gestational age (63).

Exposure to tobacco smoke in the prenatal period and/or childhood might be highly detrimental to the child’s neurodevelopment because it induces changes that alter the cell dynamics, triggering a cascade of neurotoxic risk factors that negatively affect sensory processing (64). Unhealthy activation of the neuronal nicotinic acetylcholine receptors (nAChRs) through exposure to nicotine in early childhood modulates the synaptic plasticity; most likely influences endogenous cholinergic transmissions; and alters cell, physiological, and behavioral processes in critical periods of development (65). Studies on ADHD identified some interactions between exposure to smoke in the prenatal period and specific genotypic variants, particularly affecting DAT1, DRD4, and neuronal acetylcholine receptor subunit alpha-4 (CHRNA4) (66–68). For instance, children with two copies of a DAT polymorphism exhibited greater risk of ADHD when they also had exposure to maternal prenatal smoking (69). Children with a specific allele in each DRD4 or DAT1 gene and a history of exposure to prenatal smoking in a sample of 15,000 twins from the United States exhibited a threefold higher risk of being diagnosed with ADHD. When children have specific alleles in both genes, the risk is threefold higher (67, 70).

Some evidence suggests that prenatal exposure to alcohol also causes pathological changes that increase the risk for ADHD, mainly resulting from the effect of alcohol on the modulation of the expression of the catecholamine transport system. Kim and colleagues found that prenatal exposure to ethanol in physiologically significant concentrations induced hyperactive, inattentive, and impulsive behavioral phenotypes in rats and their offspring associated with increased DAT protein expression and reduced methyl CpG-binding protein 2 (MeCP2) expression in the prefrontal cortex and striatum (71).

## Epigenetics

It is believed that the actions of the environmental factors associated with ADHD are mediated by epigenetic mechanisms (72). According to the epigenetic paradigm, even structural conditions determined by genes might be modulated, inhibited, or expressed by environmental adversities or influences. Such genomic programing directed by environmental factors is a physiological phenomenon, a type of adaptive response that begins as early as in intrauterine life. Understanding that social adversities might lead to brain structural alterations are necessary to detect vulnerabilities and formulate interventions at early neurodevelopmental stages, when the brain plasticity is greater and its modulation by means of adaptive learning is easier.

The term “epigenetics” derives from the Greek prefix “epi” and literally means, “above or beyond genetics.” As a whole, this term refers to any potentially inheritable change that might alter the expression of a gene without any direct modification of the nucleotide sequence in the DNA. It includes transient and modifiable cell alterations, as well as stable mechanisms responsible for the phenotypic identity of a given cell type (73–77). At the molecular level, the epigenetic mechanisms consist in biochemical modifications of the DNA and histone proteins – the main components of chromatin. Additional mechanisms involving interference RNA and prions also contribute to epigenetic regulation (78). The changes in the chromatin are countless and complex, including methylation of DNA cytosine–guanine dinucleotides (CpG) and post-transductional modifications of histones, including acetylation, methylation, phosphorylation, ubiquitination, and sumoylation. As a function of their chemical properties, these modifications interfere with chromatin condensation and thus modulate the access of the DNA to the transcriptional machinery (73, 78).

In the past decade, it became increasingly clear that epigenetic processes play crucial roles in the modulation of neurodevelopment. DNA methylation patterns might be influenced by factors, such as the quality of maternal care in the first years of life and exposure to maltreatment in childhood, resulting in epigenetic markers that last for life (79). In a recent study, Wong colleagues measured DNA methylation in promoter regions of three genes: DRD4, the serotonin transporter gene (SLC6A4/SERT), and the MAO-A gene. These authors analyzed that DNA samples from 46 pairs of monozygotic twins and 45 pairs of dizygotic twins (total *n* = 182) aged 5–10 years old. The results suggest that differences in DNA methylation are apparent in early childhood, even between genetically identical individuals, and that individual differences in methylation are not stable over time. That longitudinal study indicated that environmental influences behave as relevant factors in the production of individual differences in DNA methylation in the genome (80, 81).

Epigenetic changes due to intrauterine exposure might play critical roles in neurodevelopment, and maternal smoking is a significant risk factor for various adverse health outcomes in children. Joubert and colleagues investigated epigenome-wide methylation in 1062 samples of cord blood from newborns in relation to maternal smoking during pregnancy. These authors identified a set of genes with methylation changes at birth in children whose mothers smoked during pregnancy. That set included the AHRR and CYP1A1 genes, which are known to participate in the detoxification of the components of tobacco smoke through the aromatic hydrocarbon receptor signaling pathway, and gene GHI1, which had not been previously associated with the response to exposure to tobacco smoke but participates in fundamental developmental processes (82).

It is believed that the effects of the environmental factors associated with behavioral phenotypes, such as ADHD, are mediated by epigenetic changes in specific neuronal sites (72). In 2014, van Mil and colleagues sought to establish whether the methylation patterns of neuronal genes observed at birth were associated with the occurrence of ADHD symptoms at age 6 years old. Patterns of DNA methylation were assessed in cord blood samples to prospectively investigate their associations with ADHD. Global analysis of 11 genomic regions showed that lower DNA methylation levels were associated with higher ADHD symptom scores. However, the authors could not distinguish whether genetic, non-genetic intragenerational transmission, or unknown environmental factors underlay that association (83).

## Future Prospects

The studies associating epigenetics and ADHD are still in their early stages, and new research lines with multidimensional scope are needed to understand how epigenetic mechanisms intervene in the determination of behavioral phenotypes. Early life experiences, involving primary affective-regulatory processes, may become embedded in the circuitry of the developing brain through epigenetic modifications and lead to long-term changes in neurobiology and behavior. Analysis of the full genome using high-resolution methylation array (like Infinium HumanMethylation450 BeadChip array – Illumina) allows for the identification of the methylation pattern that underlies clinical phenotypic variations. Wide association of the epigenome and changes in the methylation profile might be used in the investigation of patients exhibiting symptoms regarding deficits in self-regulation and behavioral disorders, such as ADHD. The ability to screen the genome for high-resolution opens a new perspective for neurophysiological diagnosis, contributes to elucidation of the roles that epigenetic mechanisms play in the susceptibility to various emotional clinical phenotypes and might contribute to the formulation of positive behavioral interventions targeting individuals with ADHD, in addition to grounding appropriate treatments.

## Conflict of Interest Statement

The authors declare that the research was conducted in the absence of any commercial or financial relationships that could be construed as a potential conflict of interest.
